# Jejunal intussusception following distal gastrectomy with Billroth I anastomosis: a case report

**DOI:** 10.3389/fsurg.2026.1817048

**Published:** 2026-10-09

**Authors:** Jing Guo, Runjie Hou, Yuan Zhang, Kaimeng Wang, Xiaobiao Song, Jijun Wang

**Affiliations:** 1 Gastrointestinal Surgery Department, Baotou Central Hospital, Baotou, Inner Mongolia, China; 2 Inner Mongolia University of Science and Technology Baotou Medical College Central Clinical College, Baotou, Inner Mongolia, China; 3 Baotou Clinical Medical College, Inner Mongolia Medical University, Hohhot, Inner Mongolia, China

**Keywords:** adult, Billroth I, case report, gastrectomy, intussusception, surgery

## Abstract

A 41-year-old male patient presented with acute persistent abdominal pain for 4 h and was admitted to our hospital in October 2023. The patient had a height of 178 cm and a weight of 55 kg, with a precisely calculated body mass index (BMI) of 17.4 kg/m² and a body surface area of 1.73 m². He was diagnosed with acute gastric perforation and underwent emergency distal gastrectomy combined with Billroth I gastroduodenal anastomosis and intraoperative jejunal feeding tube placement. On postoperative day (POD) 19, the patient developed aggravated abdominal distension and paroxysmal abdominal pain without fever, vomiting, or diarrhea. Laboratory tests showed slightly elevated inflammatory markers and normal serum lactate, while abdominal computed tomography (CT) revealed typical intestinal intussusception and partial intestinal obstruction. Emergency re-laparotomy confirmed antegrade jejunojejunal intussusception located approximately 20 cm distal to the Treitz ligament, with an intussusception length of approximately 40 cm. Manual reduction was successfully performed without intestinal resection or intraoperative complications. The patient achieved uneventful gastrointestinal function recovery and was discharged on POD 12 after reoperation. No recurrence of intussusception was observed during 6 months of outpatient follow-up. This study reports a rare case of late-onset antegrade jejunojejunal intussusception following Billroth I gastrectomy. Combined effects of low BMI-related loose mesentery, intra-abdominal postoperative adhesions, and indwelling jejunal feeding tube were identified as potential associative risk factors rather than definitive causal factors. We aim to summarize the clinical characteristics, diagnostic strategies, and standardized prevention and treatment protocols of this rare postoperative complication to supplement relevant clinical evidence.

## Introduction

A 41-year-old male patient was admitted to our hospital on October 20, 2023, with the chief complaint of abdominal pain lasting for four hours. The patient weighed 55 kg and was 178 cm in height, with an estimated body mass index (BMI) of 17 and a body surface area of 1.73 m². He had epigastric pain with rebound tenderness and muscle stiffness. On the same day, the patient underwent gastrectomy combined with a Billroth I anastomosis and abdominal drainage. Follow-up computed tomography (CT) of the abdomen revealed a tube in the left abdominal cavity, a mass-like area with high-density regions in the center, and a feeding tube (Figure 1), raising suspicion for intussusception. An emergency laparotomy was subsequently conducted under general anesthesia. During this procedure, approximately 20 cm distal to the Treitz ligament, we observed a telescopic intussusception of the proximal jejunum that had entered the distal jejunum, giving rise to an intussusception measuring approximately 40 cm in length (Figure 2). The intestinal segment was successfully lowered without any complications during surgical process (Figure 3).

**Figure 1:**
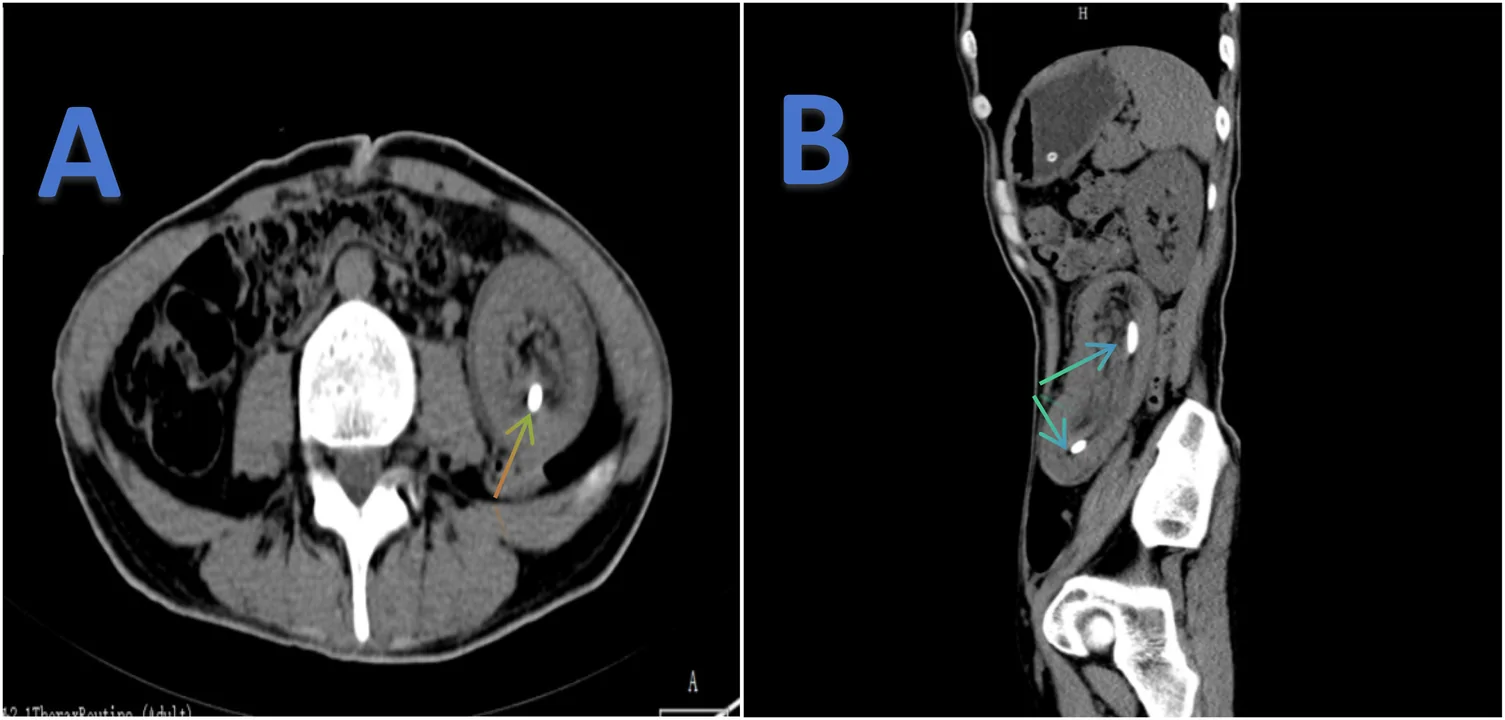
**(A)** The intestinal wall was thickened and featured the passage of nutrient tubes (yellow arrows). **(B)** Intussusception (blue arrows).

**Figure 2:**
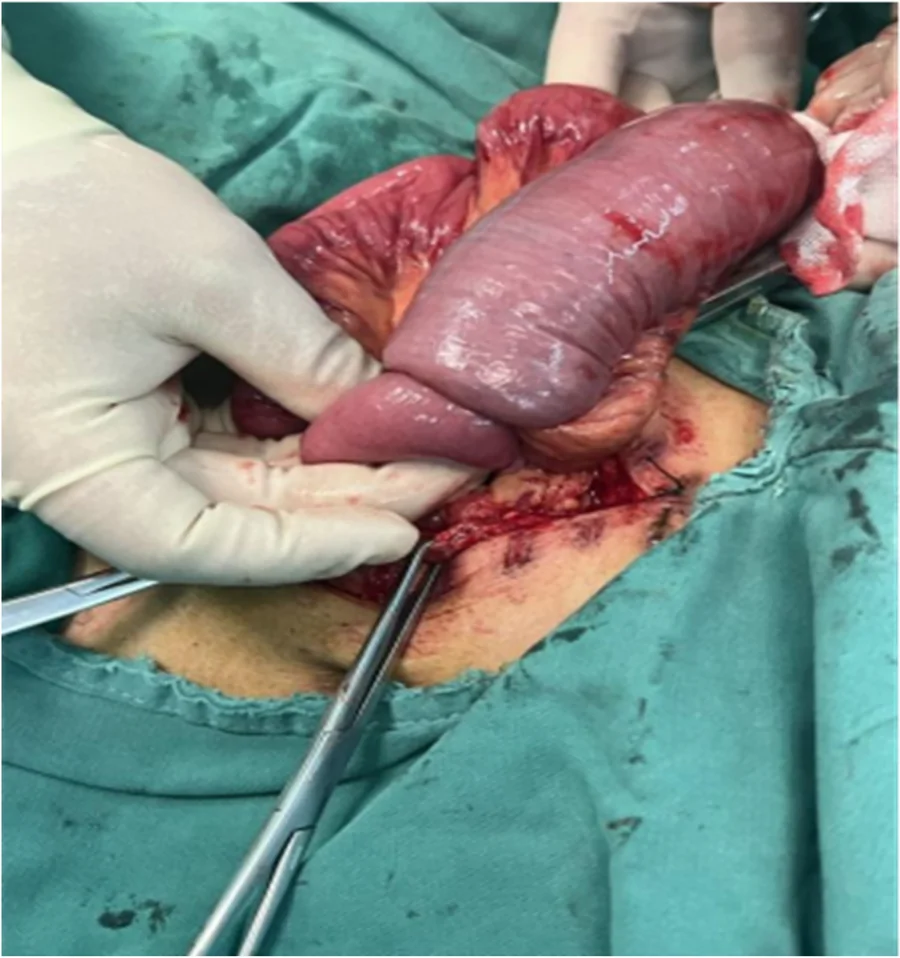
Intussusception observed during surgery.

**Figure 3:**
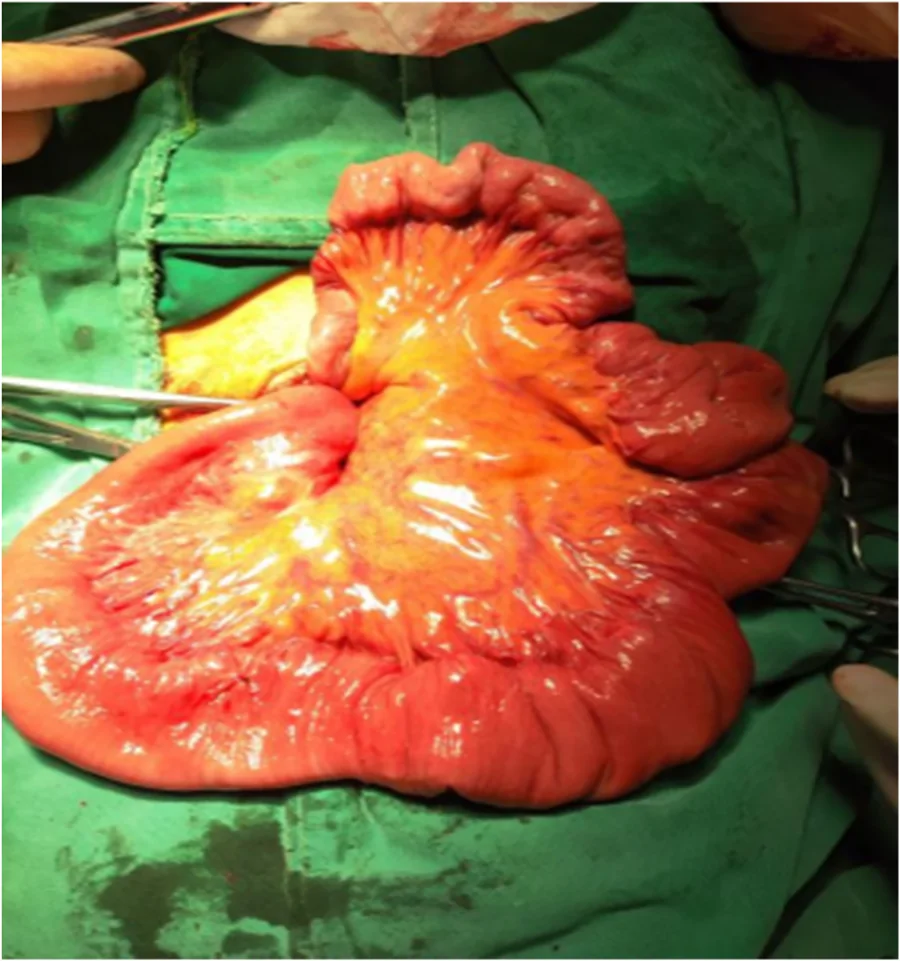
Abatement following surgical intervention.

In adults, postoperative intussusception is generally considered a rare clinical occurrence, representing 5% of all cases of intussusception (1). In most patients diagnosed with intussusception following Roux-en-Y gastric bypass (RYGB), no clear lead point is identified during surgical exploration (2).

In our current case, the intussuscepted bowel was located at the end of a jejunal feeding tube, and intra-abdominal adhesions were conspicuously dense. The development of intussusception may be associated with abdominal adhesions relating to the feeding tube, the curvature of the feeding tube, and the depth of insertion (3). Furthermore, patients diagnosed with lower BMI have a loose mesentery that is more likely to result in intussusception.

We recommend that conservative treatment be attempted initially for small bowel intussusception following gastrectomy. Emergency surgical resection should only be performed if there is evident intestinal inflammation or ischemic necrosis (4).

When implementing strategies to prevent complications associated with an enteral feeding tube, two key points must be taken into consideration. First, the feeding tube must have adequate side holes to relieve pressure and avoid local irritation in any part of the intestine. Second, the initiation rate of enteral feeding should be controlled at 10–20 mL/h (3). In addition, specific technical measures, such as fixing a 4–5 cm segment of the jejunum to the abdominal wall and minimizing the distance from the duodenojejunal flexure to the jejunal feeding stoma to 15 cm, may be further advantageous for preventing the occurrence of this complication (5).

Postgastrectomy intussusception is a rare but challenging condition for surgeons. Mortality from intussusception increases conspicuously after 48 h subsequent to gastrectomy, thus necessitating surgical intervention. Furthermore, the local resection of these portions of the intestine is required if intestinal necrosis is detected. Thus, surgeons must maintain a high degree of suspicion for postoperative intussusception when diagnosing patients to reduce the possibility of misdiagnosis.

## Data Availability

The original contributions presented in the study are included in the article/Supplementary Material, further inquiries can be directed to the corresponding author.
